# Norms and standardizations in neuropsychology via equivalent scores: software solutions and practical guides

**DOI:** 10.1007/s10072-021-05374-0

**Published:** 2021-06-17

**Authors:** Edoardo Nicolò Aiello, Emanuele Giovanni Depaoli

**Affiliations:** 1 University of Milano-Bicocca, Monza, Italy; 2 School of Medicine and Surgery, University of Milano-Bicocca, Monza, Italy; 3 Department of Statistical Sciences, University of Padua, Padua, Italy

**Keywords:** Equivalent score, Neuropsychological assessment, R, Normative data, Tolerance limits, Psychometrics

## Abstract

**Background:**

Norming neuropsychological tests and standardizing their raw scores are needed to draw objective clinical judgments on clients’ neuropsychological profile. The Equivalent Score (ES) method is a regression-based normative/standardization technique that relies on the non-parametric identification of the observations corresponding to the outer and inner tolerance limits (oTL; iTL) — to derive a cut-off, as well as to between-ES thresholds — to mark the passage across different levels of ability. However, identifying these observations is still a time-consuming, “manual” procedure. This work aimed at providing practitioners with a user-friendly code that helps compute TLs and ES thresholds.

**Methods:**

R language and RStudio environment were adopted. A function for identifying the observations corresponding to both TLs by exploiting Beta distribution features was implemented. A code for identifying the observations corresponding to ES thresholds according to a *z*-deviate-based approach is also provided.

**Results:**

An exhaustive paradigm of usage of both the aforementioned function and script has been carried out. A user-friendly, online applet is provided for the calculation of both TLs and ESs thresholds. A brief summary of the regression-based procedure preceding the identification of TLs and ESs threshold is also given (along with an R script implementing these steps).

**Discussion:**

The present work provides with a software solution to the calculation of TLs and ES thresholds for norming/standardizing neuropsychological tests. These software can help reduce both the subjectivity and the error rate when applying the ES method, as well as simplify and expedite its implementation.

## Introduction

### Background

When quantitatively assessing individuals’ neuropsychological functioning via psychometric tests, raw scores ought to be standardized in order to: (a) draw individual-level clinical judgments; (b) *intra*−/*inter*-individually compare performances that differ in nature and metrics [20]. For raw scores to be standardized, normative values have to be inferred first from a healthy population sample [10]. Neuropsychological data often do not meet distributional assumptions — mostly due to high *inter*-individual variability and ceiling/floor effects [1, 13, 17]: non-parametric approaches are thus to be preferred when drawing norms [10]. Moreover, controls for inferential errors are needed when classifying a performance as either “normal” or defective [4].

The Equivalent Score (ES) method [5, 6, 19] standardizes regression-adjusted [14] scores into a 5-point ordinal scale that allows drawing clinical judgments: ESs = 0 and 1 meaning “defective” and “borderline,” respectively; ES = 2 meaning “low-end normal”; ESs = 3 and 4 meaning “normal.” A cut-off is identified through the outer one-sided non-parametric lower tolerance limit (oTL) — i.e., the highest ascending-order-ranked adjusted scores yielding a safety level *p* ≥ .95 that no more than 5% of the population performs below it. The method thus inferentially addresses a performance as impaired if falling within the “worst” 5% of the normative sample, by keeping the risk of drawing a wrong inference below 5%. To control for inferential errors, the inner one-sided non-parametric lower TL (iTL) is also computed — i.e., the lowest observation yielding a safety level *p* ≥ .95 that at most 95% of the population performs above it. Adjusted scores lower than the oTL and greater than the median are classified as ES = 0 and 4, respectively. Between-ES conversion thresholds (0 → 1; 1 → 2; 2 → 3; 3 → 4) are identified by subdividing into three equal segments the range of adjusted scores between the oTL and the median via a *z-*score-based approach.

### Aims

The ES method is representative of regression-based approaches to norm/standardize neuropsychological tests by non-parametrically drawing cut-offs and controlling for inferential errors [11, 20]. It is the most widely used neuropsychometric approach in Italy [2, 3]. Although whatever statistical software (e.g., R [15]) that builds in linear regressions allows identifying predictors that raw scores should be adjusted for [4], to compute TLs and ES thresholds is still currently a time-consuming, “manual” procedure. Indeed, the computerized way to identify TLs recently proposed by Capitani and Laiacona [6] is still based on a trial-and-error procedure (which exploits an online calculator provided by Casio [7]). Moreover, to the best of the authors’ knowledge, no software solutions are currently available to calculate ES thresholds.

The present work thus provides practitioners with an R-based, user-friendly guide and software solution for computing TLs and ESs thresholds.

## Methods

R language (3.6.2) and RStudio environment were selected for writing the code since they are widely used and freely accessible [15, 16].

**Fig. 1:**
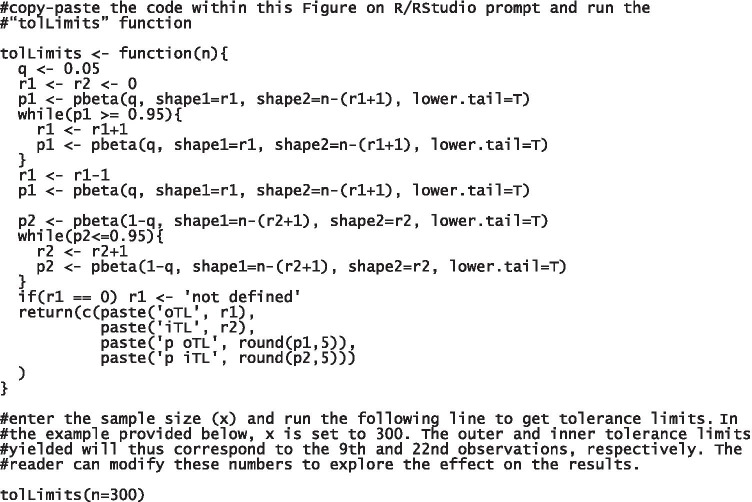
An R function to computer inner and outer tolerance limits (tolLimits). Notes. The programming lines allow to get the observations corresponding to both the outer and the inner tolerance limits along with respective safety levels. All code lines are divided in blocks to facilitate the inspection. Useful descriptions are reported in those lines introduced by a hashtag. Instructions: (1) run the first line; (2) enter the sample size (x) in the last line; (3) run the last line to get the observations corresponding to tolerance limits along with respective safety levels

**Fig. 2:**
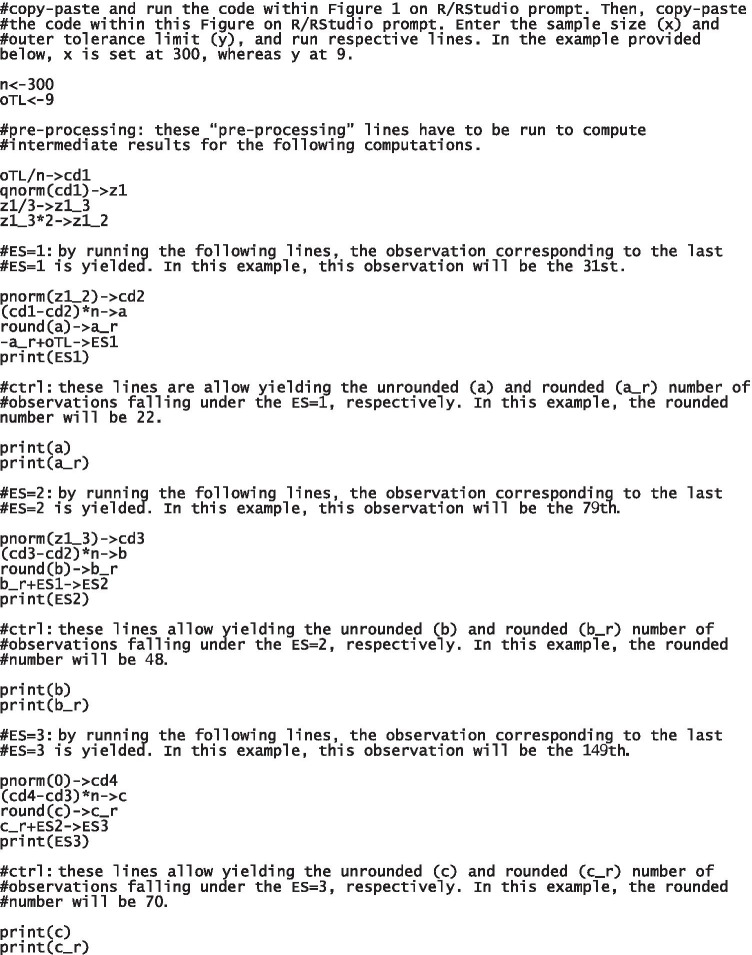
An R script to compute Equivalent Scores (ESs) thresholds. Notes. The programming lines allow to get the observations corresponding to the last Equivalent Scores (ESs) = 1, 2, and 3. All code lines are divided in blocks to facilitate the inspection. Useful descriptions are reported in those lines introduced by a hashtag. Instructions: (1) enter the sample size (x) and outer tolerance limit (y) and run respective lines; (2) run #pre-processing lines; (3) run #ES1 lines: by running print(ES1) line, the observation (r_*i*_) corresponding to the last ES = 1 is yielded; (4) run a and a_r to get the unrounded and rounded number of *r*_*i*_s falling under the ES = 1, respectively. These “control” lines (#ctrl) are useful to determine whether the unrounded number of *r*_*i*_s is close to the rounding threshold (.5; e.g., 25.47): this allows users to judge whether a should be rounded up or down (indeed, round() function by default rounds up numbers to the nearest integer when decimals are ≥ .5). If deciding to round up a, the last ES = 1 will be equal to ES1+1; therefore, +1 will have to be added to the b_r+ES1->ES2 line. Steps (3) and (4) are to be repeated on the following lines in order to get the last ES = 2 and 3. Users have to note that the applet associated with this script automatically rounds up number to the nearest integer when decimals are ≥ .5 (according to the round() function)

The level of R proficiency required to perform the calculations below and get the results is limited to (a) downloading R (https://cran.r-project.org) and RStudio (http://www.rstudio.com/), (b) copying and pasting the scripts in the syntax section of RStudio and subsequently (c) running them according to the instructions provided in Figs. 1 and 2, and (d) reading the results in the output section of RStudio.

The script is mathematically adherent to the procedures described by Capitani and Laiacona [6] for identifying TLs and ES thresholds [5, 6, 19].

TLs were computed separately from ES thresholds. A function (tolLimits) yielding the observations corresponding to the outer and inner TLs along with their exact safety levels (*p*) was implemented by adapting the iterative procedure described by Capitani and Laiacona [6] (Fig. 1). tolLimits allows calculating the observations corresponding to both TLs and respective *p*s from a given sample size *N*.

Standard steps to identify ES thresholds from oTL and *N* are then provided (Fig. 2):

1. First, the cumulative density (cd1) corresponding to the oTL is computed as oTL/n;
2. The *z*-deviate at cd1 is subsequently calculated via qnorm() and then divided by 3. The three segments comprising observations that will fall within the ES = 1, 2, and 3 are bounded by the following *z*-deviates, respectively: z1_3 (i.e., the quotient of z1/3), z1_2 (i.e., the double of z1_3), and 0;
3. The observation corresponding to the last ES = 1 is identified by adding the number of observations comprised within the oTL to that of observations falling under the ES = 1. While the former addend is equal to the oTL itself, the latter is identified by the following: (1) computing the cumulative density at z1_2 (cd2) via pnorm(); (2) multiply by *N* the subtraction of cd1 from cd2 (a); (3) adding the rounded a to the oTL. The same procedure is applied in order to identify the last ES = 2 and 3, with the only exception that the second addend corresponds to the number of observations falling under the ES = 1.

## Results

A paradigm of usage of the scripts displayed in Figs. 1 and 2 is provided. The paradigm takes into account both categorical (e.g., *sex*) and continuous (e.g., years of *age* and *education*) predictors and is run by assuming that the following steps have already been implemented [4]:

1. Testing the effect of each categorical/continuous predictor on the criterion via linear model (LM) analyses (e.g., independent samples *t*-test for *sex*; simple linear regression for *age* and *education*);
2. Identifying for each predictor the transformation that best fits the shape of its relationship with the criterion — e.g., ln(100-*age*) and √(*education*) — i.e., the transformed predictor which yields the highest *R*^2^/*β*;
3. Entering predictors that independently have the largest effect into a stepwise multiple regression procedure in order to identify the best model — i.e., the one comprising only significant predictors. The *p* value can be in this case Bonferroni-corrected — *p*_adjusted_ = .05/*k* with *k* being the number of predictors [19].
4. Adjusting raw scores (with the exception of those corresponding to either the minimum or the maximum of the test) via the following equation yielded by the best model: AS = RS + [−*b*_1_ * (*x*_1_ − *M*_*x*1_)] + [−*b*_2_ * (*x*_2_ − *M*_*x*2_)]… + [−*b*_*i*_ * (*x*_*i*_ − *M*_*xi*_)]; if *sex* is a significant predictor, the term + [−*b*_*sex*_ * (*sex* − .5)] can be added (with 0 = *male* and 1 = *female*);
5. Ranking adjusted scores and ordering observations in ascending order by adjusted score ranks.

An R script for the implementation of the aforementioned steps is made available at https://github.com/enaiello/ES_ENA_EGD.

Let *N* = 300 be the sample size. tolLimits function will thus yield the following results: oTL = 9 with *p* = .964 and iTL = 22 with *p* = .954. The oTL and the iTL will be thus equal to the adjusted scores corresponding to the 9th and 22nd observations, respectively. Let the range of the test 0–15; let oTL and iTL be = 4.571 and 5.203, respectively. Adjusted scores will be assigned an ES = 0 if 4.571≤; the cut-off will thus be 4.572. Adjusted scores between the oTL and the iTL (4.572 ≤ adjusted scores ≤5.203) will lay in the so-called gray area [6] — i.e., when drawing clinical judgments based on these adjusted scores (i.e., whether a performance is impaired or not), one cannot be sure that the error risk is kept at 5%.

If the oTL falls within a run of equal adjusted scores — i.e., adjusted scores belonging to the same rank (tied ranks) — the highest observation below the “formal” oTL can be regarded as the “actual” oTL in order to keep the error risk 5%<. Similarly, if the iTL falls within a run of tied-ranked adjusted scores, the lowest observation above the “formal” oTL can be regarded as the “actual” oTL.

The *z*-deviate corresponding to the oTL (z1) is -1.880794 (given a cumulative density cd1 of .03). z1_3 and z1_2 are then computed and = -.6269312 and -1.253862, respectively. The number of observations being attributed an ES = 1 is 22 (a_r) — since the corresponding unrounded number (a) is -22.4838. The last ES = 1 will be thus the 31st observation — i.e., the cumulative number of observations from the lowest to this.

Similar computation will yield the number of observations falling under the ES = 2 and 3 — 48 and 70, respectively — as well as observations corresponding to the last ES = 2 and 3 — the 79th and the 149th, respectively. Let the adjusted scores corresponding to the last ES = 1, 2, and 3 be = 5.897, 7.285, and 9.771, adjusted scores being assigned an ES = 1, 2, 3, or 4 fall within the ranges displayed in Table 1.

**Table 1:** Tolerance limits (TLs) and Equivalent Score ranges for putative adjusted scores on a test

Outer TL	Inner TL	Equivalent Scores				
		0	1	2	3	4
4.571	5.203	≤ 4.571	4.572–5.897	5.898–7.285	7.286–9.771	≥ 9.772

If ES thresholds happened to fall within a run of equal adjusted scores, the same rule adopted with respect to the iTL can be applied (regarding the lowest observation above the “formal” ES threshold as the “actual” threshold). This expedient would make the considered ES area wider and thus allows being as conservative as possible when passing from one ES to the next (i.e., to assign a higher level of ability to an adjusted score).

tolLimits function and the script for computing ESs thresholds have been also implemented as user-friendly, online applets by means of the R package *shiny* [8] (retrievable at https://egdp.shinyapps.io/tolLimits/). The applet yields both TLs and the observations corresponding to the last ES = 1, 2, and 3 by simply entering the sample size.

## Discussion

The present work provides practitioners in the neuropsychometric field a software solution (R scripts and user-friendly, online applets) for computing TLs and ES thresholds to norm neuropsychological tests, as well as for implementing regression-based steps that precede their calculation — this allowing to simplify and expedite the norming procedure, as well as to make it less subjected to “human” errors.

This last assertion is especially relevant when taking into account socio-demographic changes that give rise to the need of updating neuropsychological test norms [18]. Indeed, several neuropsychological tests still rely on normative data collected decades ago [2, 3, 19].

This article can be thus regarded as a “software translation” of the original ESs approach [5, 6, 19]. However, a single note should be made with regard to the median: here, it is addressed as falling within the ES = 4, according to the most recent statement by Capitani and Laiacona [6].

Moreover, practitioners should bear in mind that modifications can be adopted within the regression-based procedure preceding the calculation of TLs and ESs thresholds. First, transforming predictors can be complemented with polynomial regression analyses — which may help identifying the actual shape of the relationship between the predictor and the outcome and thus select the best transform [9]. Second, in order to select the best predictors, methods other than addressing *R*^2^/*β* statistics can be adopted (see Heinze et al. [12] for a comprehensive review on the topic).

In the present work, two computational issues regarding the ES method have also been approached: (a) attributing the number of observations within each ES in uncertainty scenarios (i.e., when a non-integer, close-to-rounding-threshold number is yielded); (b) defining TLs and ES threshold in the presence of tied ranks [19]. However, practitioners have to be aware of the fact that the above proposals are merely empirical, “thumb” rules. Indeed, while the former aspect is mostly subjected to each practitioner’s judgment, future works are needed to provide theoretical support to the latter.

Finally, it should be highlighted that the most relevant contribution of this work to users is arguably represented by the user-friendly applet (https://egdp.shinyapps.io/tolLimits/). This applet indeed allows to immediately get the observations corresponding to both TLs (iTL; oTL) and ES thresholds (last ES = 1, 2, and 3) by simply (a) accessing the link and (b) entering the sample size.

## Data Availability

The complete R script described in the present work is openly retrievable on GitHub at https://github.com/enaiello/ES_ENA_EGD. A user-friendly applet to compute TLs and ESs thresholds based on the present R script is accessible at https://egdp.shinyapps.io/tolLimits/.
